# Focal pulsed-field ablation for recurrent para-Hisian premature ventricular contractions after failed radiofrequency ablation: A case report

**DOI:** 10.1016/j.hrcr.2026.06.017

**Published:** 2026-06-22

**Authors:** Xianjin Hu, Wenjie Li, Bangjiaxin Ren, Aobo Gong, Mingyang Tang, Rui Zeng

**Affiliations:** 1 Department of Cardiology, West China Hospital, Sichuan University, Chengdu, Sichuan, China; 2 Department of Cardiology, Chengdu Fifth People’s Hospital, Chengdu, Sichuan, China

**Keywords:** Pulsed-field ablation, Para-Hisian, Premature ventricular contractions, Junctional rhythm, Atrioventricular block, Right bundle branch block

**Key Teaching Points**

• Focal pulsed-field ablation demonstrates feasibility and potential safety for treating recurrent para-Hisian premature ventricular contractions after failed radiofrequency ablation, offering a promising alternative in this high-risk anatomical region where thermal energy poses significant risk of permanent conduction system injury.
• The transient appearance of junctional rhythm and even third-degree atrioventricular block during pulsed-field ablation delivery may not necessarily indicate irreversible damage but should still be cautiously interpreted as possible early signs of His bundle involvement, prompting immediate procedural pause and close monitoring.
• Persistent right bundle branch block postprocedure underscores the multifactorial nature of conduction disturbances, potentially involving both mechanical trauma and electroporative effects, highlighting the importance of preprocedural counseling on potential procedural complications.

## Introduction

Premature ventricular contractions (PVCs) originating from the para-Hisian region present a therapeutic challenge due to the proximity of the cardiac conduction system and the risk of inadvertent atrioventricular (AV) block. Conventional radiofrequency ablation (RFA) in this region is often limited by safety concerns, resulting in suboptimal efficacy or recurrence. Focal pulsed-field ablation (PFA), a novel non-thermal modality with tissue selectivity, offers potential safety advantages in high-risk anatomical locations. We report a case of recurrent para-Hisian PVCs following failed RFA, successfully treated with focal PFA. We also discuss the implications of transient junctional rhythm and AV block observed during energy delivery, highlighting PFA as a feasible strategy for arrhythmias near the His bundle.

## Case report

A 41-year-old woman without structural heart disease presented with a 6-month history of disabling palpitations. 24-hour Holter monitoring demonstrated a 38.8% PVC burden (Supplementary 1-A). She had undergone RFA 3 months prior at another institution, where electroanatomic mapping localized the origin to the para-Hisian region. However, the PVC burden remained unchanged (33.5%) at the 1-month follow-up (Supplementary 1-B). On admission, the 12-lead electrocardiogram (ECG) suggested a para-Hisian origin with a QRS duration of approximately 140 milliseconds (positive QRS complexes in leads II, III, and aVF; a QS pattern in lead V1; an M-shaped morphology in lead V2; positive QRS complexes with notching on the ascending limb in leads I and aVL; Supplementary 2-A).

**Figure 1:**
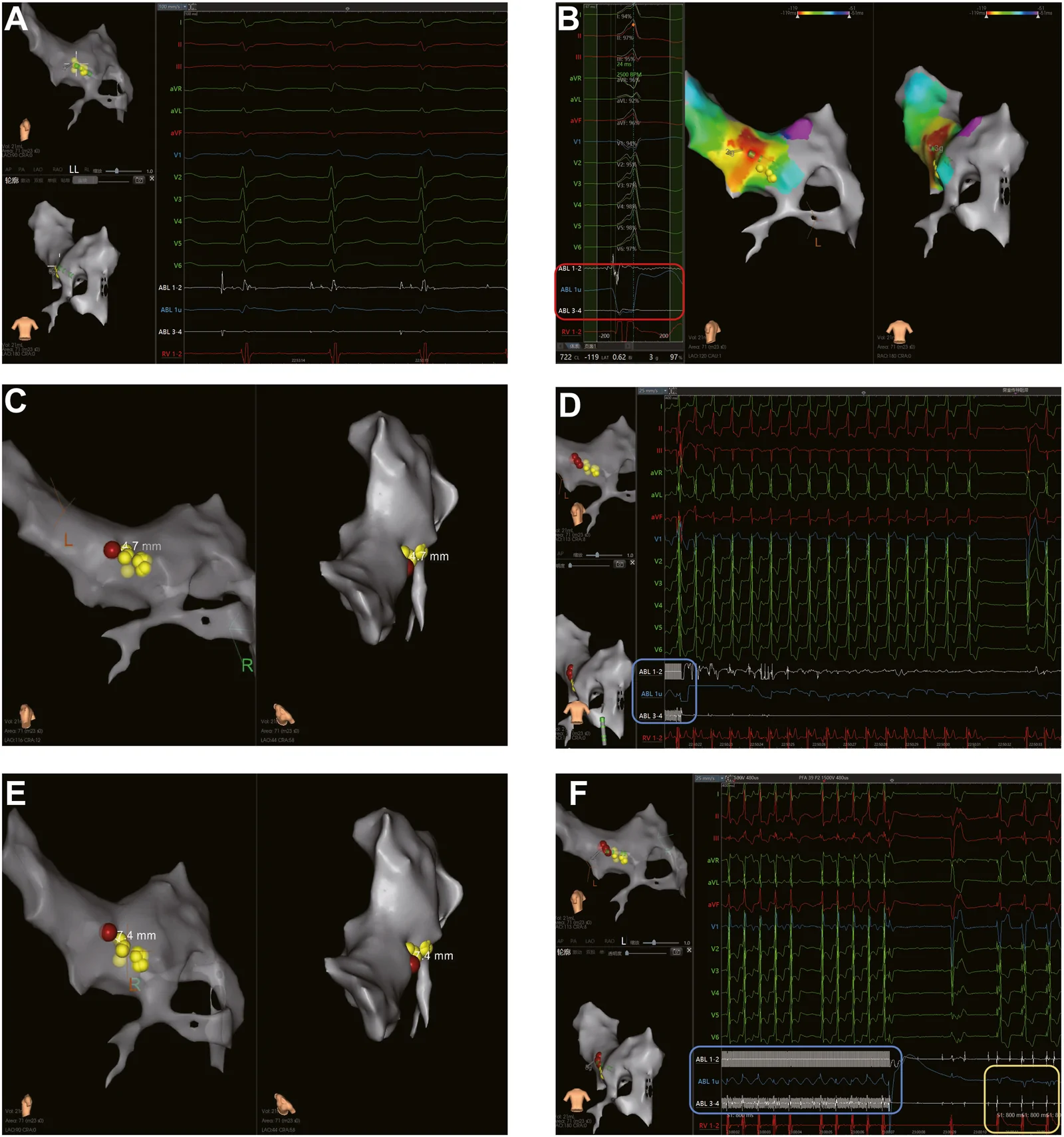
Focal pulsed-field ablation of para-Hisian premature ventricular contractions. Yellow markers indicate sites where His bundle electrograms were recorded; red markers denote ablation applications; and the red box highlights the unipolar electrogram. **A-B:** Electroanatomic mapping localized the earliest activation to the para-Hisian region. **C:** Ablation target site located 4.7 mm from the maximal His potential. **D:** Junctional rhythm with atrioventricular dissociation immediately after pulsed-field ablation (PFA). Blue boxes indicate PFA pulse artifacts on the ABL 1–2 and 3–4 channels. **E:** Consolidation ablation 7.4 mm from the His bundle. **F:** Transient third-degree atrioventricular block lasting 72 seconds before recovery. Blue boxes highlight PFA pulse artifacts on the ABL 1–2 and 3–4 channels. Yellow boxes indicate ventricular pacing artifacts at a cycle length of 800 milliseconds.

Electroanatomical mapping was performed using the LEAD-Mapping™ system (Sichuan Jinjiang Electronic Medical Device Technology Co., Ltd., Chengdu, China). Procedural mapping of the para-Hisian region identified the earliest activation site at the septal band of the right ventricular crista supraventricularis. The local unipolar electrogram exhibited a pure QS morphology preceding the surface QRS by 24 milliseconds, confirming a focal ventricular origin (Figure 1A, 1B; yellow tags denote His bundle potentials; red area indicates earliest activation zone; red box highlights the unipolar electrogram). During mapping of this region, a right bundle branch block (RBBB) was induced by mechanical catheter contact before any energy delivery. Ablation was performed using a microsecond bipolar, biphasic focal PFA catheter (Sichuan Jinjiang Electronic Medical Device Technology Co., Ltd.). Each application consisted of 5 pulse trains with voltage titrated to the relatively medium setting (1.5 kV, effective energy delivery duration: 480 μs). A total of 4 PFA applications were delivered. To prioritize safety in the para-Hisian region, the first 2 applications were performed at a further distance from the optimal target, resulting in no conduction disturbance or PVC reduction. The third application was delivered at a target site located 4.7 mm from the maximal His potential (Supplementary 3-A; Figure 1C). Immediately upon energy delivery, a junctional rhythm emerged, followed by the onset of AV block, characterized by a non-conducted P wave followed by a ventricular escape beat (Figure 1D). This episode persisted for 44 seconds before the restoration of 1:1 AV conduction (PR interval 198 ms; Supplementary 3-B). A fourth consolidation application was delivered 7.4 mm from the maximal His potential (Figure 1E), resulting in transient third-degree AV block (Figure 1F). During this period, right ventricular pacing was delivered at a cycle length of 800 milliseconds. Complete AV conduction recovered after 72 seconds (PR interval 187 ms; Supplementary 3-C). Acute success was confirmed by non-inducibility under isoproterenol and a 20-minute waiting period (Supplementary 2-B). Given the immediate and complete suppression of PVCs upon ablation at this site, further mapping of the left ventricular outflow tract or aortic root was not pursued. The predischarge ECG showed sinus rhythm with a PR interval of 174 milliseconds (Supplementary 2-C). At the 1-month follow-up, the patient remained free of PVCs with a 0% burden confirmed by 24-hour Holter monitoring (Supplementary 4) and a preserved PR interval (172 ms; Supplementary 2-D). At the 6-month follow-up, a 12-lead ECG confirmed the continued absence of PVCs (Supplementary 2-F). Finally, at the 8-month follow-up, a repeated 24-hour Holter monitor demonstrated a complete absence of PVCs with a 0% overall burden (Supplementary 5).

## Discussion

This case illustrates the successful application of focal PFA for para-Hisian PVCs, supporting its viability for arrhythmias adjacent to the His bundle. The observed junctional rhythm and transient AV block during energy delivery warrant careful interpretation.^1^^,^^2^ Although these phenomena may indicate effective lesion formation, they must be provisionally regarded as warning signs of conduction system interaction. Emerging clinical data corroborate these observations. A recent case series demonstrated that PFA delivered to the septal RVOT at a distance of approximately 15 mm from the His bundle achieved a 98% reduction in PVC burden at 12 months with intact AV conduction.^3^ Conversely, closer proximity mandates caution. In a prospective cohort, transient complete AV block was similarly observed during para-Hisian PFA at 1600 V, resolving within 50 seconds.^4^ A recent report of slow pathway ablation using focal-bipolar PFA at 1400 V near the His bundle also demonstrated successful target elimination accompanied by transient AV block.^5^ This closely mirrors our experience.

Mechanistically, preclinical data indicate that PFA effects on the His bundle are voltage-dependent within a specific range (600–1800 V). Higher voltages prolong conduction recovery time, but Masson staining reveals structurally preserved His bundle cells.^6^ Consistent with this, in a canine model targeting the interventricular septum, specialized conduction tissue and Purkinje fibers within the ablation lesions maintained intact ultrastructure and nuclei.^7^ This contrasts with the contraction band necrosis typically observed in PFA-ablated myocardium.^8^ However, the safety margin of PFA near the AV node appears highly dependent on both the specific electric field parameters and the total number of applications. Recent experimental data from the PFA-CONDUCT study demonstrated that direct focal PFA at the porcine AV junction initially caused a transient AV block that recovered, whereas a sustained, irreversible AV block consistently occurred after a third application.^9^ This cumulative injury effect is consistent with our clinical observation, where spatial titration and limiting applications at the nearest target resulted in only reversible conduction stunning. Although cryoablation is a well-established, safe alternative utilizing reversible cryomapping, focal PFA may offer a comparable safety profile. Conceptually, low-voltage PFA creates a zone of reversible electroporation, functioning as an electrical mapping strategy analogous to cryomapping while potentially providing greater lesion durability. Compared with conventional radiofrequency ablation, exploratory ablation using low-voltage PFA may induce large-scale reversible electroporation effects,^10^^,^^11^ thereby offering greater procedural tolerance and safety in such high-risk anatomical regions.

Notably, the RBBB persisted at the 8-month follow-up. Retrospective review confirmed its onset during catheter manipulation before PFA delivery (Supplementary 3-D), pointing to an initial mechanical etiology. However, its long-term persistence suggests a complex, multifactorial injury mechanism. The subsequent PFA applications likely exacerbated the pre-existing mechanical stunning. Furthermore, the precise anatomical origin of the arrhythmia cannot be definitively confirmed without left-sided mapping; however, the chosen right para-Hisian ablation target is inherently associated with a high risk of right bundle branch injury. This site-specific anatomical vulnerability, combined with the acute mechanical trauma and the prior radiofrequency substrate, likely drove the progression to a permanent RBBB. This additive effect suggests that PFA is relatively tissue-selective; however, its safety margin may be significantly narrowed in the presence of pre-existing fibrosis and acute procedural trauma. Additionally, the transient 44-second AV block observed in our case indicates direct electric field involvement of the conduction axis. Although 1:1 AV conduction was preserved at 6 months, the potential for subclinical damage progressing to late-onset conduction disturbances over the patient’s lifespan remains unknown. Extended monitoring is essential to fully rule out delayed effects on the His-Purkinje system.

## Conclusion

Focal PFA represents a potential supplementary option for para-Hisian PVCs, particularly in cases refractory to conventional radiofrequency ablation. Although pulsed-field energy is relatively tissue-selective, the absolute safety margins in high-risk anatomical regions remain undefined. The persistent RBBB at 6 months highlights the risk of permanent conduction damage, which likely resulted from a cumulative effect of a prior radiofrequency substrate, acute mechanical trauma, and PFA. Furthermore, the transient AV block recovered in this case; however, the risk of late-onset AV block over the long term remains unknown. Prospective studies with extended follow-up are necessary to determine the definitive safety, optimal dosing, and late-onset conduction risks of PFA near the AV conduction system.

## Funding Sources

This work was supported by the 10.13039/501100004829Science and Technology Department of Sichuan Province (Grant Number: 2024YFFK0046), 1∙3∙5 Project for Disciplines of Excellence-Clinical Research Incubation Project, 10.13039/501100013365West China Hospital, Sichuan University (Grant Number: 2023HXFH002). The funders had no role in data collection and analysis, decision to publish, or preparation of the manuscript.

## Disclosures

The authors declare no competing interests.

## Footnotes

Appendix Supplementary data associated with this article can be found in the online version at https://doi.org/10.1016/j.hrcr.2026.06.017.

## References

[1] Zeng R., Li F., Jiang J. (2024). The safety and feasibility of pulsed-field ablation in atrioventricular nodal re-entrant tachycardia: first-in-human pilot trial. JACC Clin Electrophysiol.

[2] Shen C., Bai R., Jia Z. (2025). Unexpected transient atrioventricular block and slow junctional rhythm using pulsed field ablation for slow pathway modification: excited or cautious for ablators. Heart Rhythm.

[3] Martignani C., Massaro G., Spadotto A. (2026). Pulsed field ablation for the treatment of ventricular arrhythmias using a focal, contact-force sensing catheter: A Single-Center case series and review. J Cardiovasc Dev Dis.

[4] Cai C., Ai Z., Wang J. (2026). Pulsed field ablation for idiopathic premature ventricular complexes: evaluation of acute and chronic lesion characteristics with cardiac magnetic resonance imaging. J Interv Card Electrophysiol.

[5] Zarębski Ł., Futyma P. (2026). Focal-bipolar pulsed field ablation as a bailout strategy for radiofrequency-resistant atrioventricular nodal reentrant tachycardia. J Interv Card Electrophysiol.

[6] Zhai Z., Wang Y., Shi L., Liu X. (2024). Impact of pulsed electric field ablation on His bundle conduction: A preclinical canine study. Med Sci Monit.

[7] van Zyl M., Ladas T.P., Tri J.A. (2022). Bipolar electroporation across the interventricular septum: electrophysiological, imaging, and histopathological characteristics. JACC Clin Electrophysiol.

[8] Nakagawa H., Farshchi-Heydari S., Maffre J. (2024). Evaluation of ablation parameters to predict irreversible lesion size during pulsed field ablation. Circ Arrhythm Electrophysiol.

[9] Linz D., Hertel J.N., Chaldoupi S.M. (2026). Monopolar biphasic focal pulsed field ablation directly at the atrioventricular junction and from within the noncoronary cusp: the PFA-CONDUCT study. Heart Rhythm.

[10] Cespón-Fernández M., Pannone L., Sieira J. (2026). Feasibility of reversible electroporation mapping in human atrial flutter. Heart Rhythm.

[11] Nakagawa H., Sugawara M., Saliba W.I., Hussein A.A. (2024). Pulsed-field ablation of AV nodal re-entrant tachycardia: are we shooting the AV node?. JACC Clin Electrophysiol.

