# Supplementary material for: Focal pulsed-field ablation for recurrent para-Hisian premature ventricular contractions after failed radiofrequency ablation: A case report
Source: HeartRhythm Case Rep. 2026 Jun 22;12(9):1148–51. doi: 10.1016/j.hrcr.2026.06.017 (PMC13593549; doi:10.1016/j.hrcr.2026.06.017)
Supplement: Supplementary file 1 [file mmc1.pdf]

A

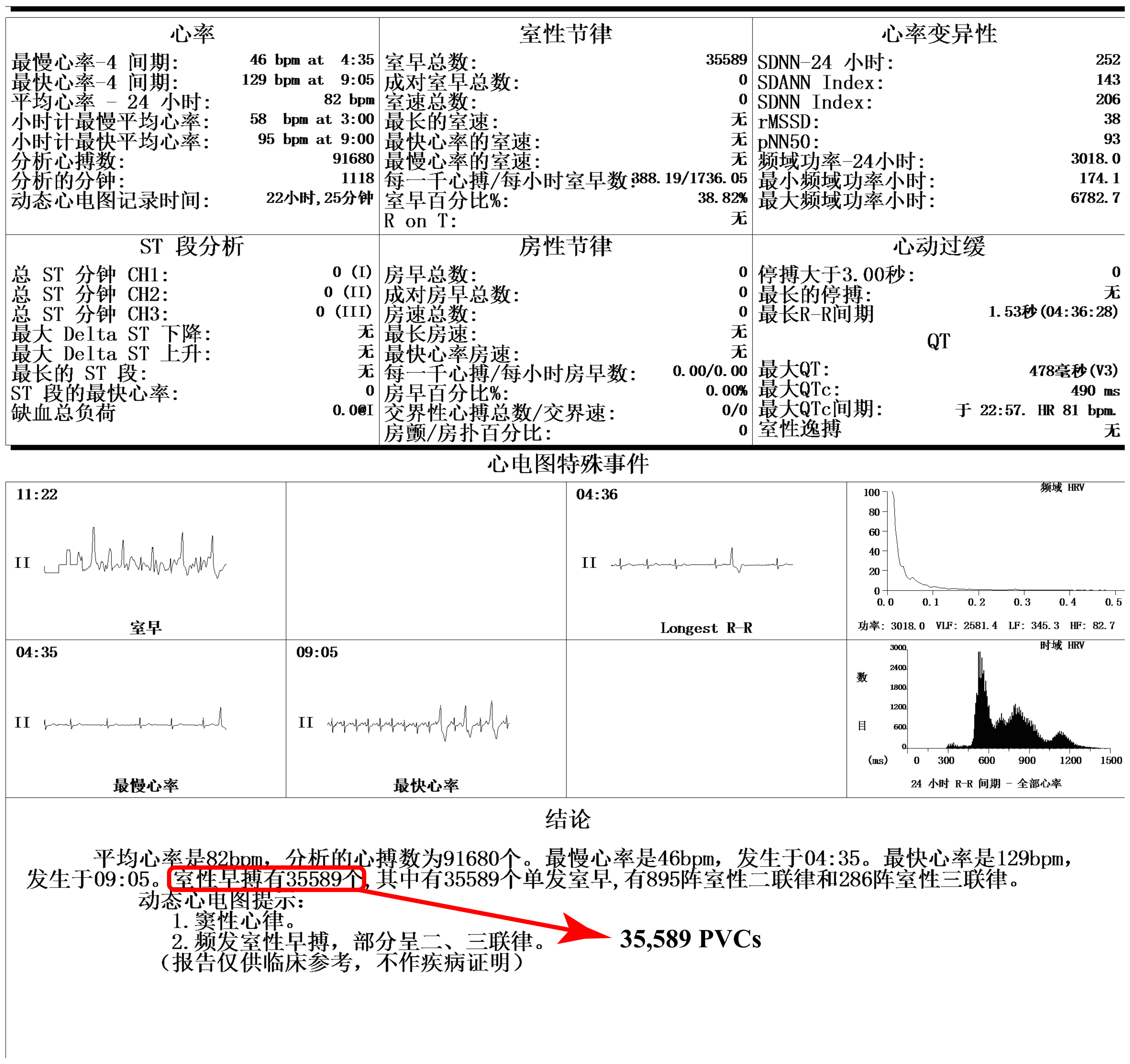
结论
平均心率是82bpm，分析的心搏数为91680个。最慢心率是46bpm，发生于04:35。最快心率是129bpm，发生于09:05。室性早搏有35589个,其中有35589个单发室早,有895阵室性二联律和286阵室性三联律。
动态心电图提示:
1. 窦性心律。
2. 频发室性早搏，部分呈二、三联律。
（报告仅供临床参考，不作疾病证明）
35,589 PVCs

A

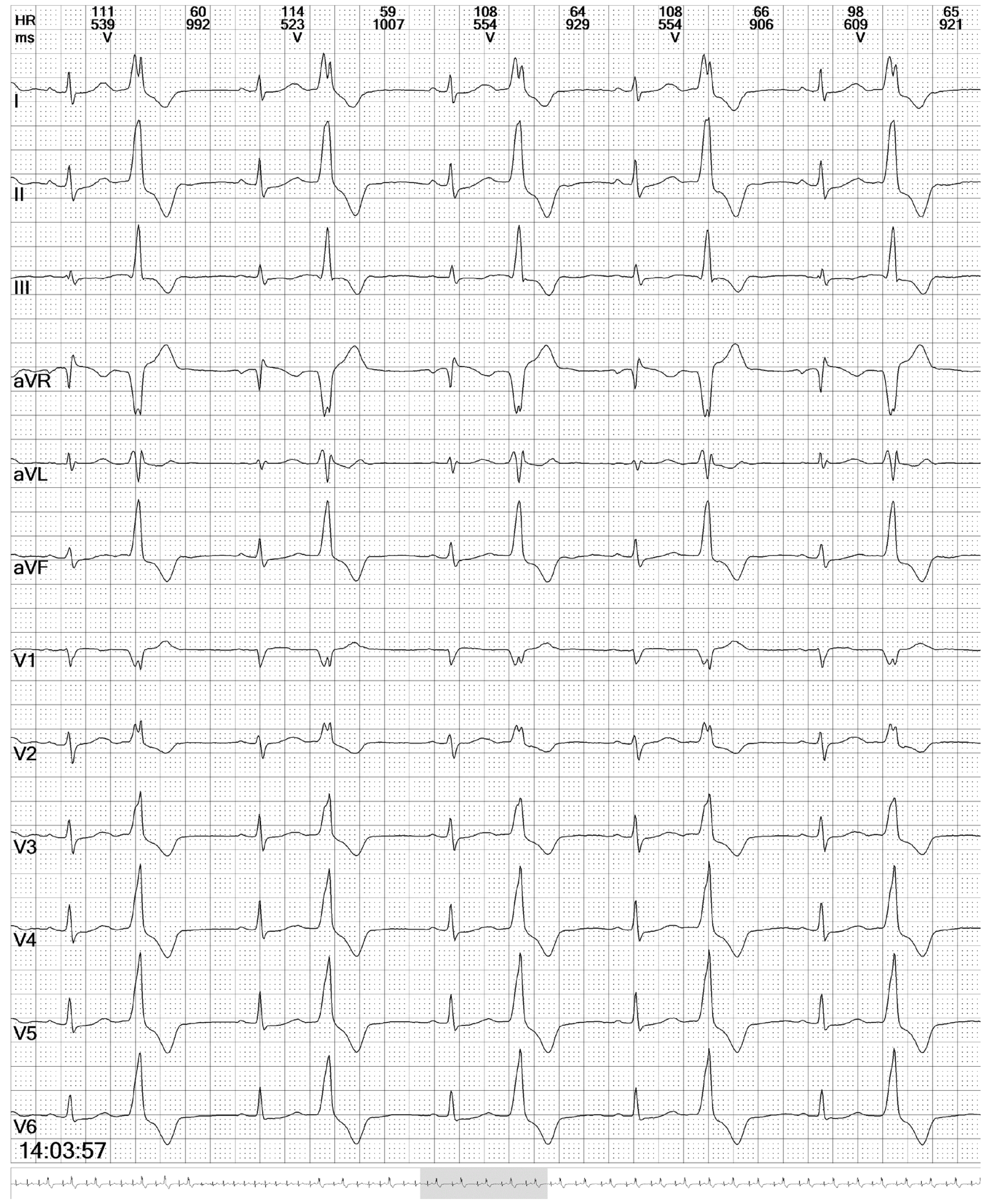
HR
ms
I
II
III
aVR
aVL
aVF
V1
V2
V3
V4
V5
V6
14:03:57

B

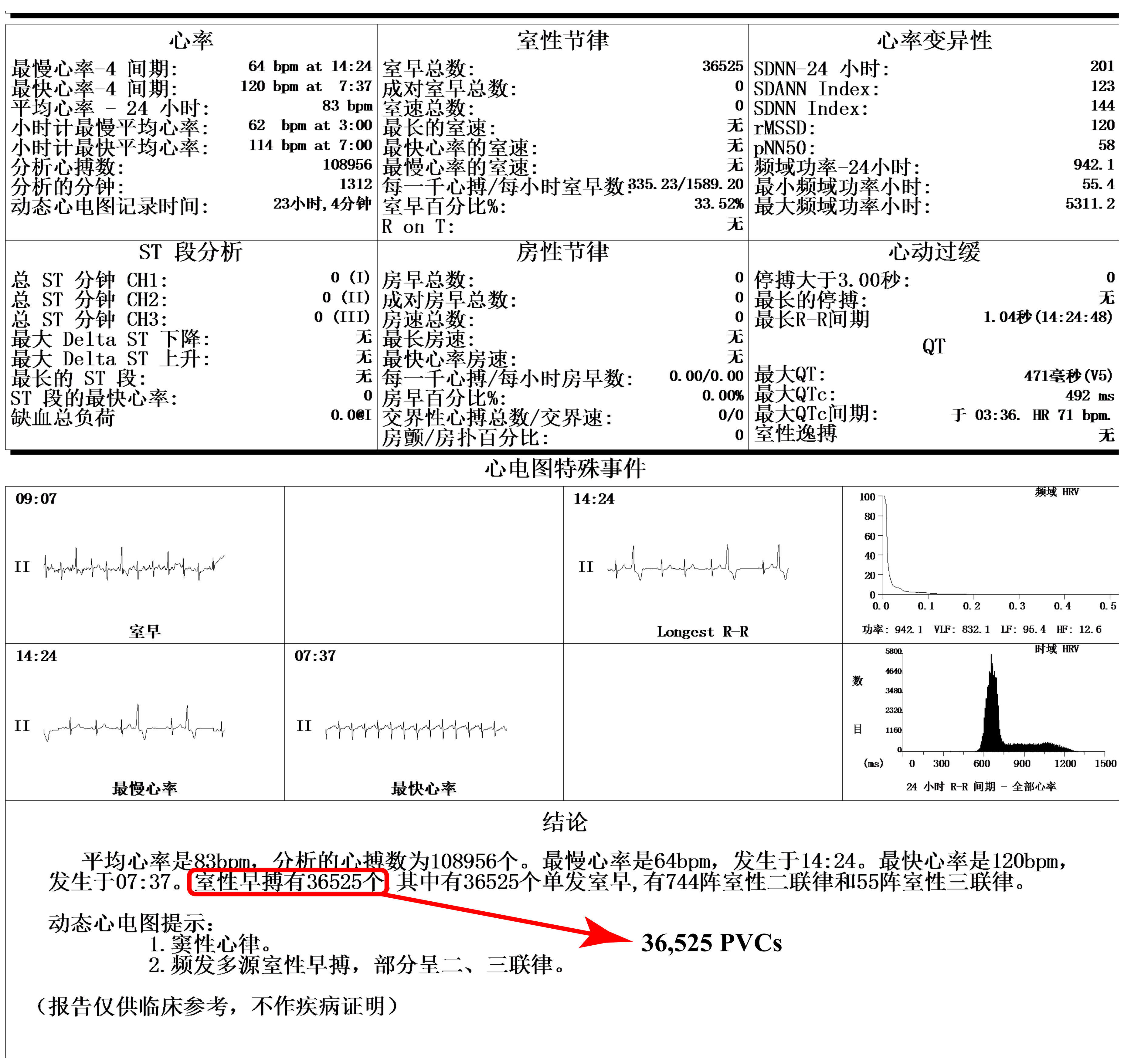
结论
平均心率是83bpm，分析的心搏数为108956个。最慢心率是64bpm，发生于14:24。最快心率是120bpm，发生于07:37。室性早搏有36525个,其中有36525个单发室早,有744阵室性二联律和55阵室性三联律。
动态心电图提示:
1. 窦性心律。
2. 频发多源室性早搏，部分呈二、三联律。
（报告仅供临床参考，不作疾病证明）
36,525 PVCs

B

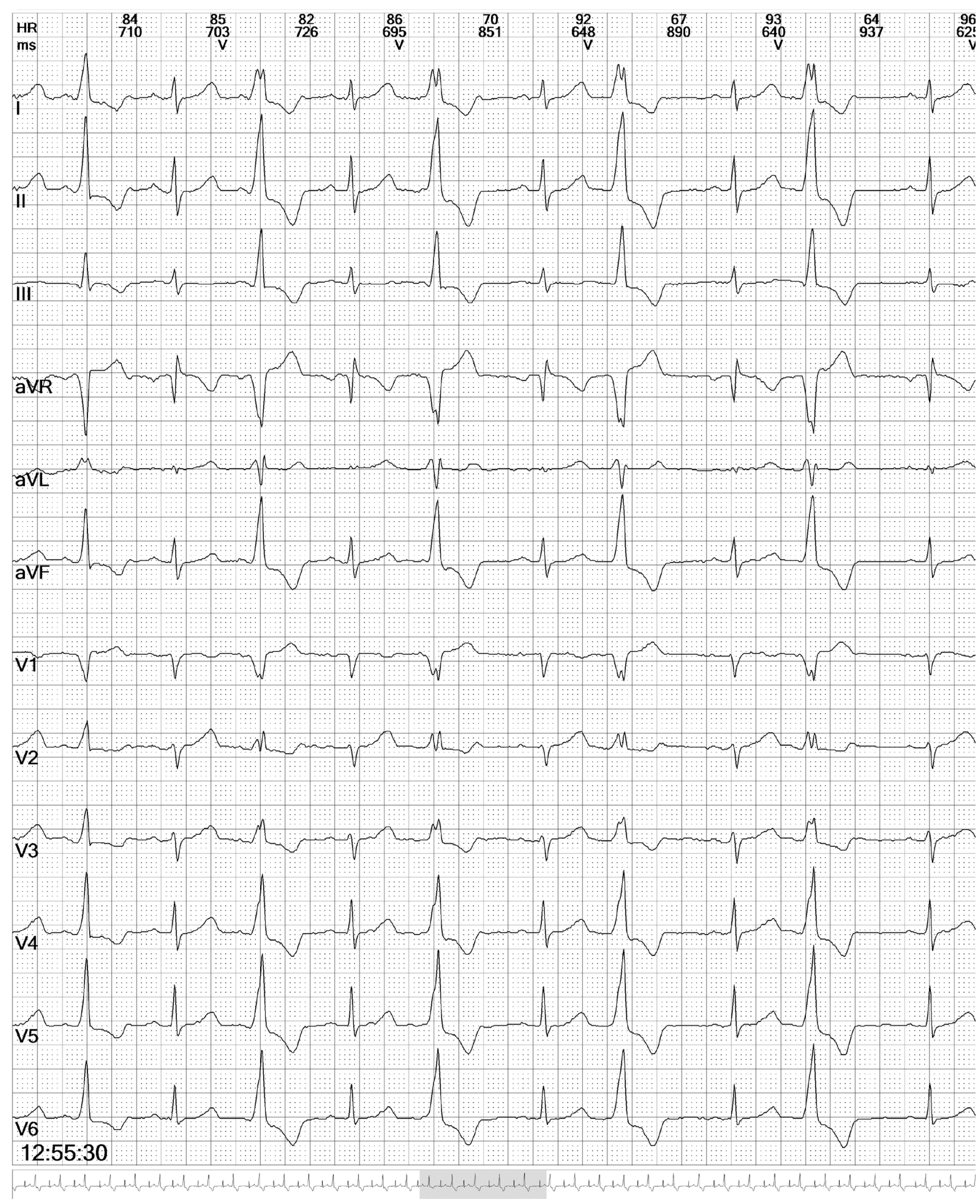
HR
ms
I
II
III
aVR
aVL
aVF
V1
V2
V3
V4
V5
V6
12:55:30
