# Supplementary material for: Focal pulsed-field ablation for recurrent para-Hisian premature ventricular contractions after failed radiofrequency ablation: A case report
Source: HeartRhythm Case Rep. 2026 Jun 22;12(9):1148–51. doi: 10.1016/j.hrcr.2026.06.017 (PMC13593549; doi:10.1016/j.hrcr.2026.06.017)
Supplement: Supplementary file 2 [file mmc2.pdf]

A

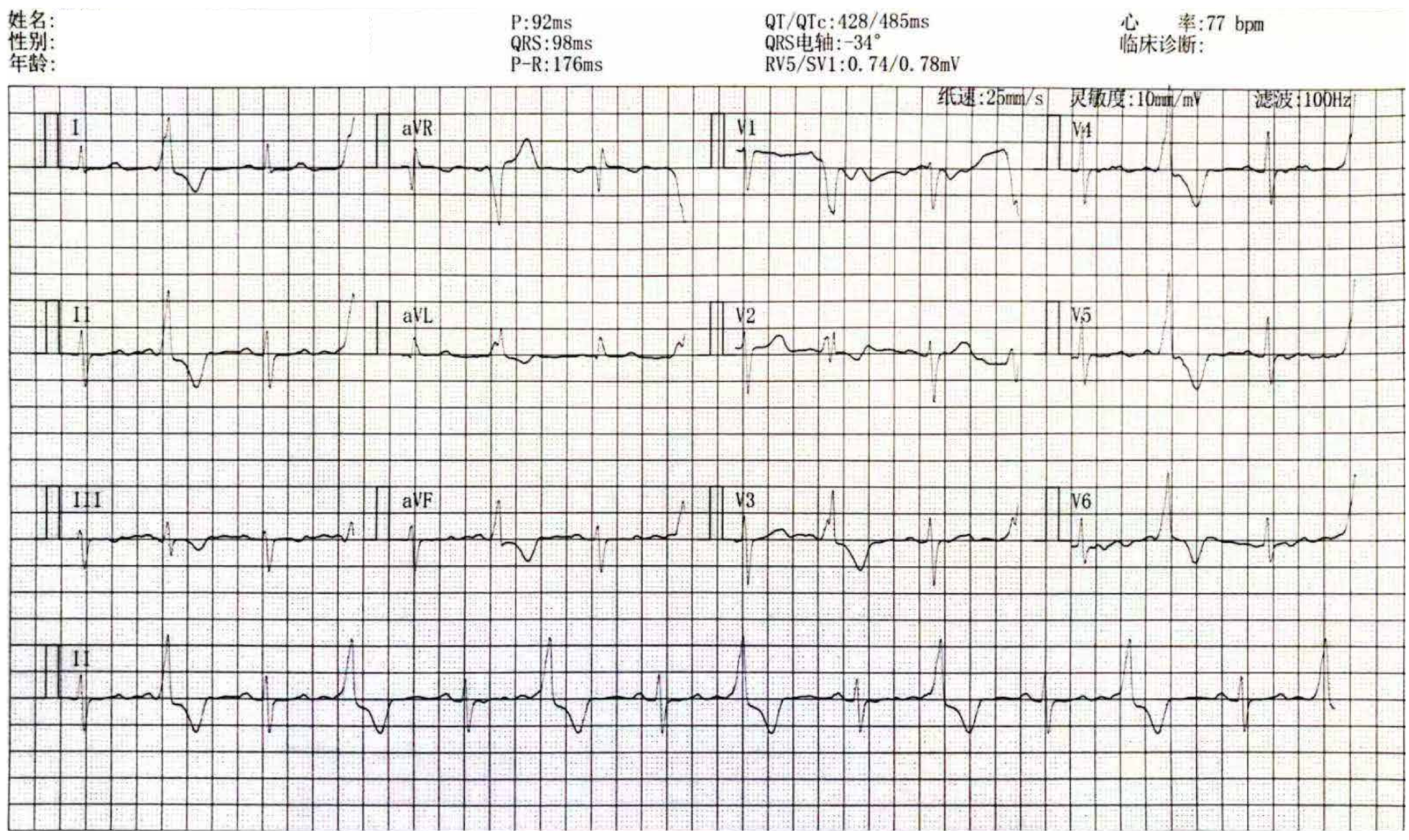
姓名:
性别:
年龄:
P:92ms
QRS:98ms
P-R:176ms
QT/QTc:428/485ms
QRS电轴:-34°
RV5/SV1:0.74/0.78mV
心 率:77 bpm
临床诊断:
纸速:25mm/s
灵敏度:10mm/mV
滤波:100Hz

B

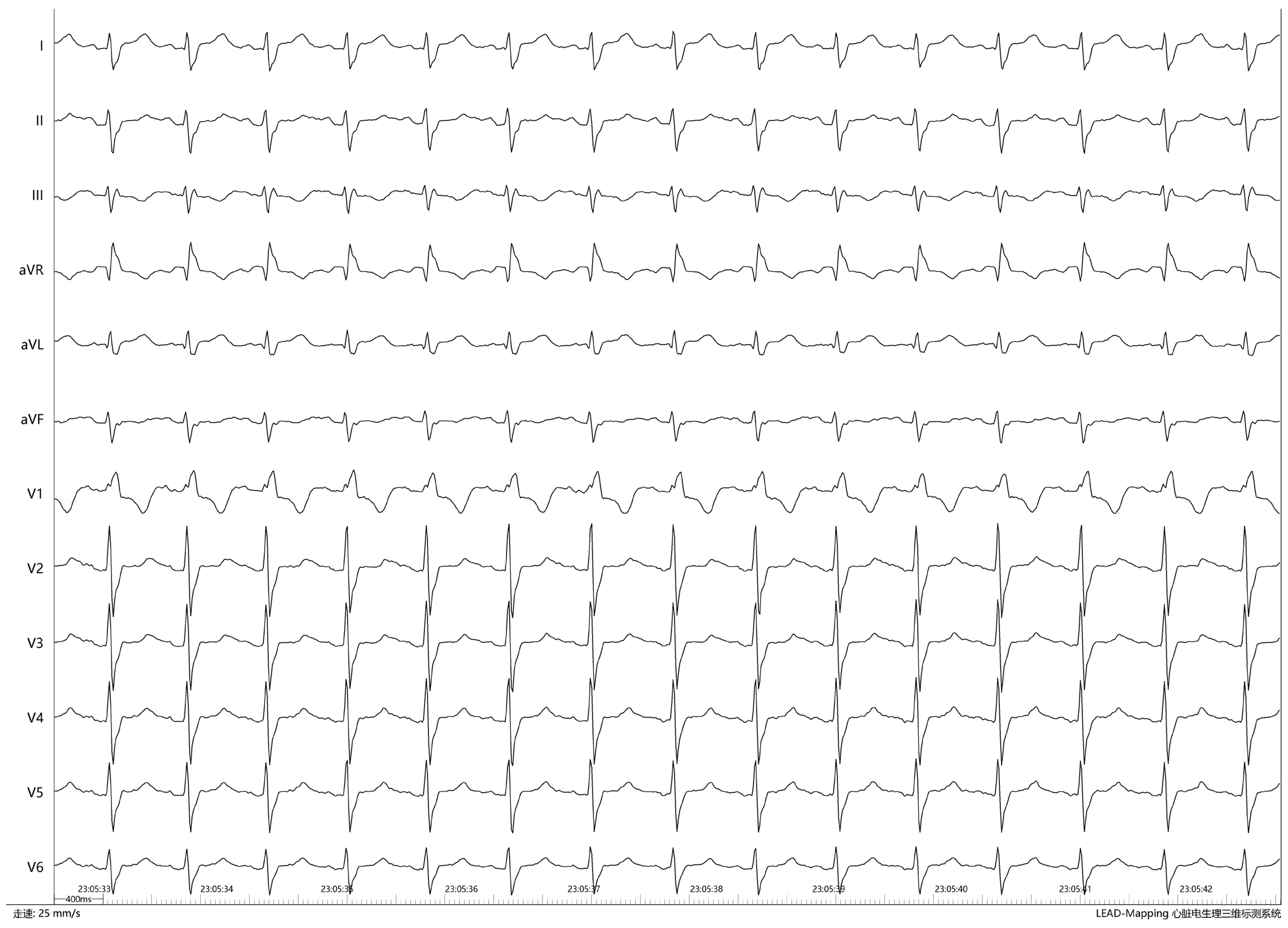
23:05:33
23:05:34
23:05:35
23:05:36
23:05:37
23:05:38
23:05:39
23:05:40
23:05:41
23:05:42
400ms
走速: 25 mm/s
LEAD-Mapping 心脏电生理三维标测系统

C

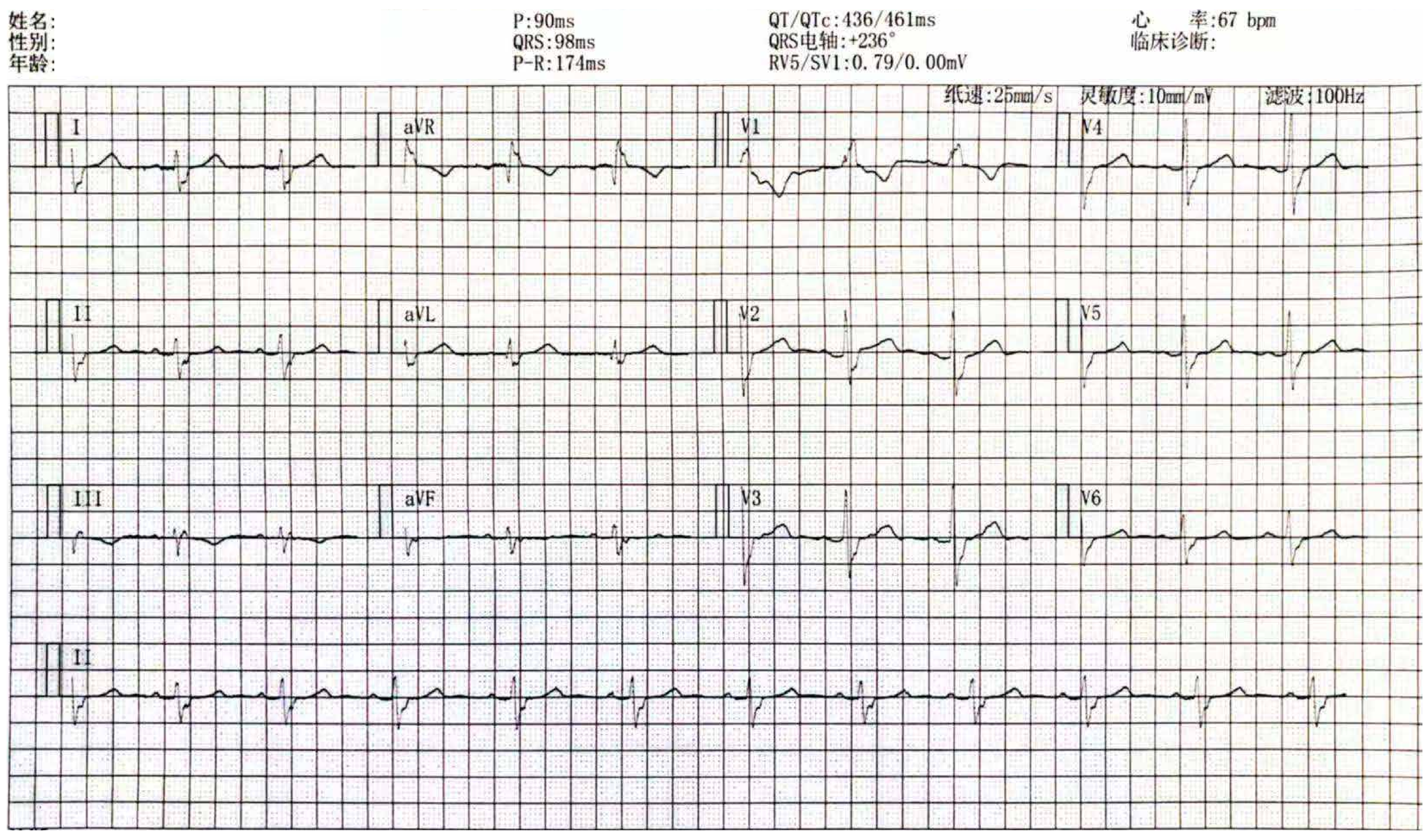
姓名:
性别:
年龄:
P:90ms
QRS:98ms
P-R:174ms
QT/QTc:436/461ms
QRS电轴:+236°
RV5/SV1:0.79/0.00mV
心 率:67 bpm
临床诊断:
纸速:25mm/s
灵敏度:10mm/mV
滤波:100Hz

D

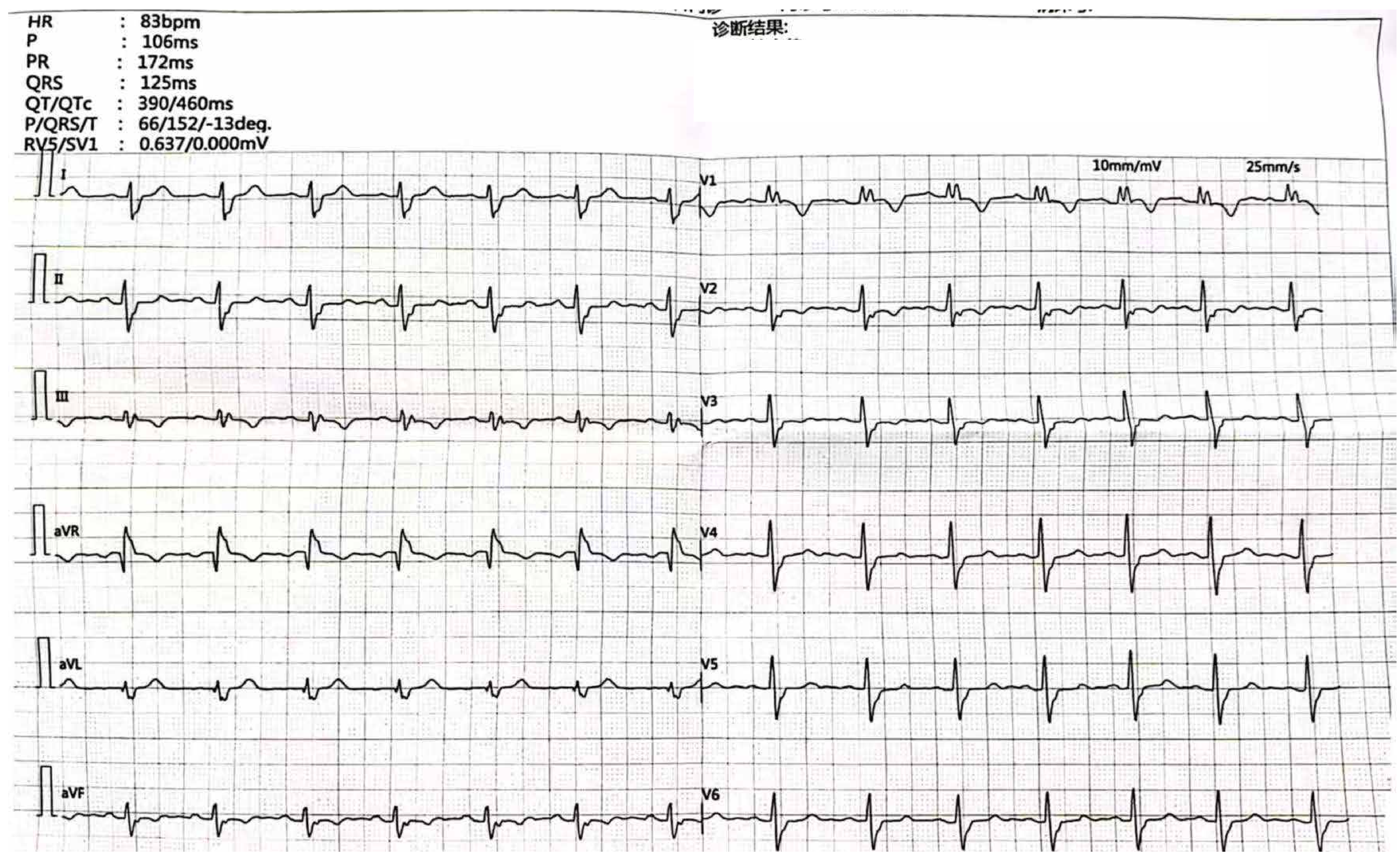
HR : 83bpm
P : 106ms
PR : 172ms
QRS : 125ms
QT/QTc : 390/460ms
P/QRS/T : 66/152/-13deg.
RV5/SV1 : 0.637/0.000mV
诊断结果:
10mm/mV
25mm/s

F

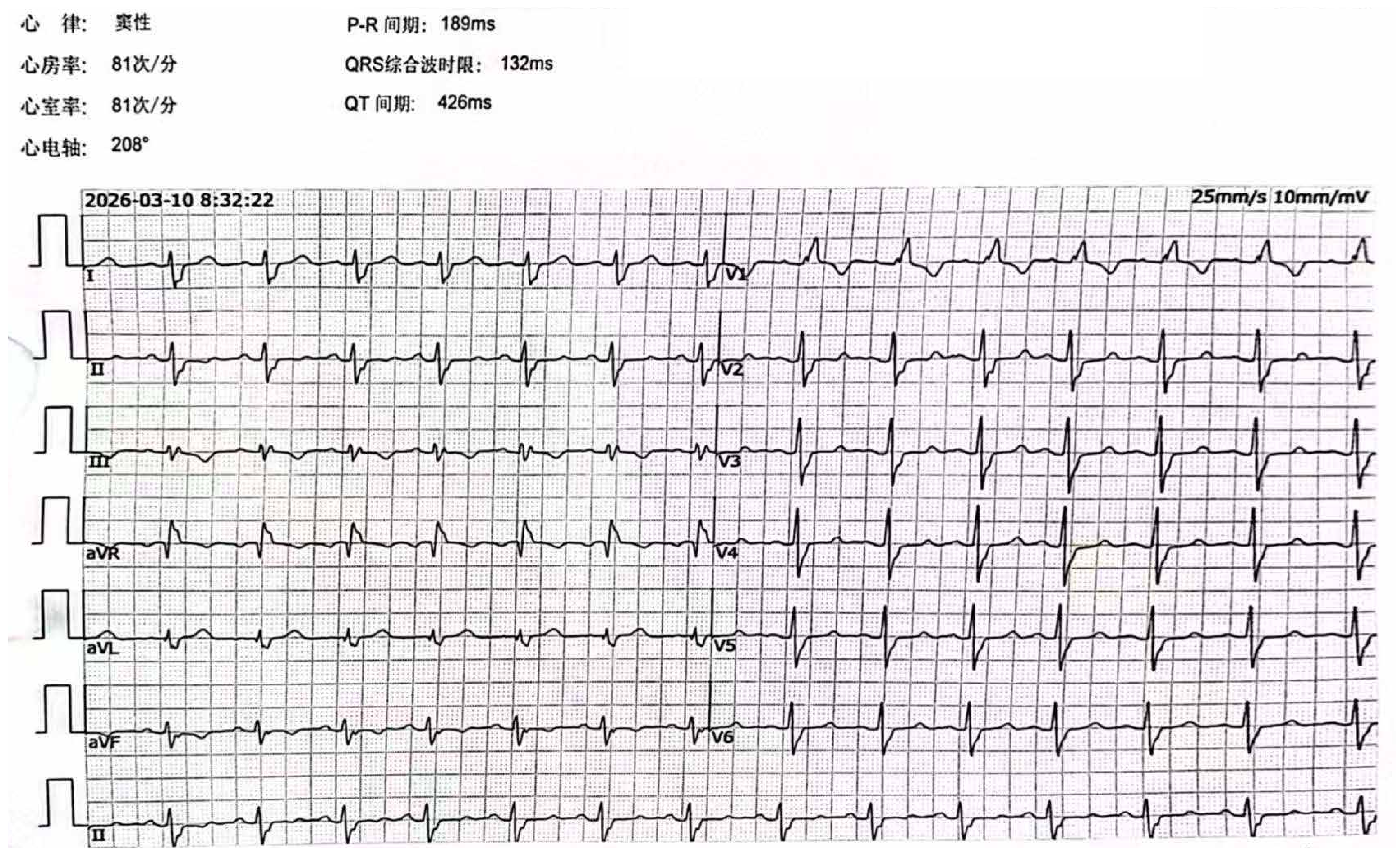
心 律: 窦性
心房率: 81次/分
心室率: 81次/分
心电轴: 208°
P-R 间期: 189ms
QRS综合波时限: 132ms
QT 间期: 426ms
2026-03-10 8:32:22
25mm/s 10mm/mV
