# Supplementary material for: Focal pulsed-field ablation for recurrent para-Hisian premature ventricular contractions after failed radiofrequency ablation: A case report
Source: HeartRhythm Case Rep. 2026 Jun 22;12(9):1148–51. doi: 10.1016/j.hrcr.2026.06.017 (PMC13593549; doi:10.1016/j.hrcr.2026.06.017)
Supplement: Supplementary file 3 [file mmc3.pdf]

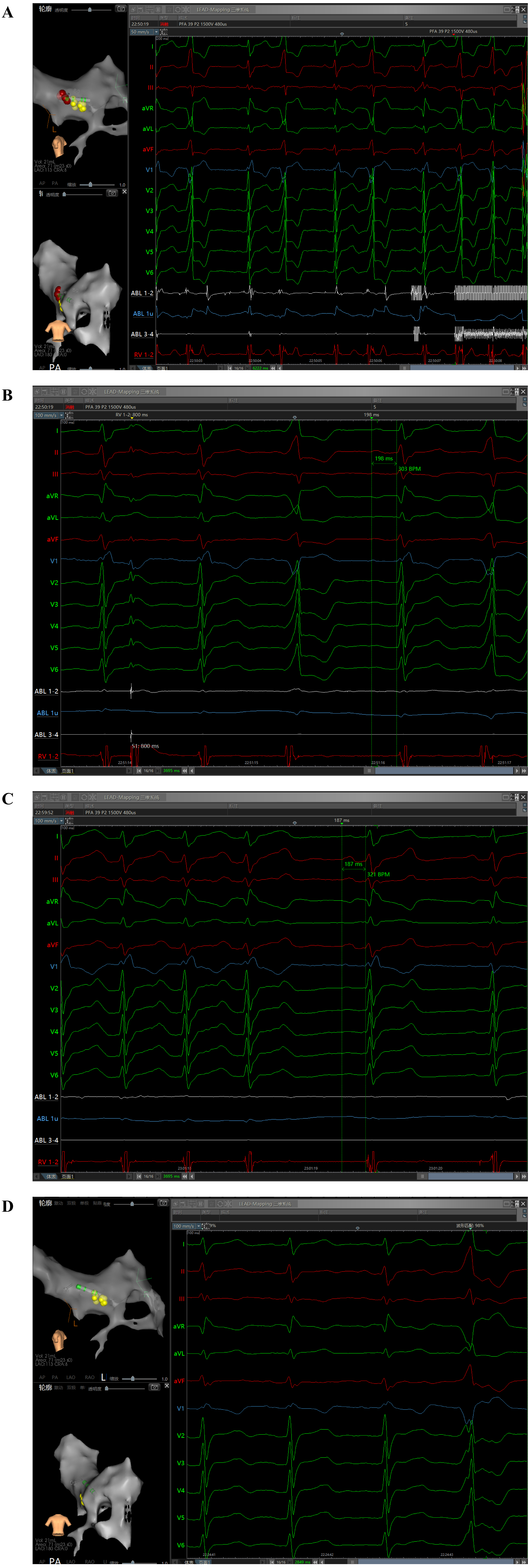
A
B
C
D
LEAD-Mapping 三维系统
PFA 39 P2 1500V 480us
I
II
III
aVR
aVL
aVF
V1
V2
V3
V4
V5
V6
ABL 1-2
ABL 1u
ABL 3-4
RV 1-2
RV 1-2: 800 ms
198 ms
303 BPM
S1: 800 ms
187 ms
321 BPM
轮廓
PA
