# Supplementary material for: Focal pulsed-field ablation for recurrent para-Hisian premature ventricular contractions after failed radiofrequency ablation: A case report
Source: HeartRhythm Case Rep. 2026 Jun 22;12(9):1148–51. doi: 10.1016/j.hrcr.2026.06.017 (PMC13593549; doi:10.1016/j.hrcr.2026.06.017)

动态心电图报告

检查信息

姓名:	性别:	年龄:	科室:
床位:	住院号:	门诊号:	记录时间:
记录时长:23小时56分钟	记录有效时长:23小时56分结束时间:2025-10-18		起搏器:N

Recording Duration: 23 h 45 min

分析统计

心搏及心率

总心搏数:106822次 Total Beats　　RR间期>2.0秒,共0次　　最长RR间期:1.369秒(10-18 05:00:59)

平均心率:76次/分 Mean HR　　最快心室率:108次/分(10-17 10:06:29)Max HR　　最慢心室率:52次/分(10-18 04:54:04)Min HR

窦性心律

心搏总数:106822次(100%)

平均心率:76次/分　　最快心率:108次/分(10-17 10:06:29)　　最慢心率:52次/分(10-18 04:54:04)

房性心律

房早总数:0

室性心律

室早总数:0 PVCs

室速:0

心率变异性

SDNN:113ms	SDANN:102ms	SDNNIndex:49ms	rMSSD:29ms	pNN50:8.65%
LF/HF:1.92	三角指数:35.19	LF:582	HF:302	

报告结论 Conclusions

1、窦性心律，不完全性右束支阻滞：总心搏数106822次；最快心率108次/分(10-17 10:06:29),最慢心率52次/分(10-18 04:54:04),平均心率76次/分；

2、最长RR间期1.369s（10-18 05:00:59）；

3、未见心律失常；

4、ST-T：ST-T未见明显异常；

5、心率变异性正常。

1. Sinus rhythm with incomplete right bundle branch block. Total beats: 106,822, maximum heart rate: 108 bpm, minimum heart rate: 52 bpm, mean heart rate: 76 bpm.
2. Longest R-R interval: 1.369 s.
3. No arrhythmias detected.
4. No significant ST-T abnormalities.
5. Normal heart rate variability.

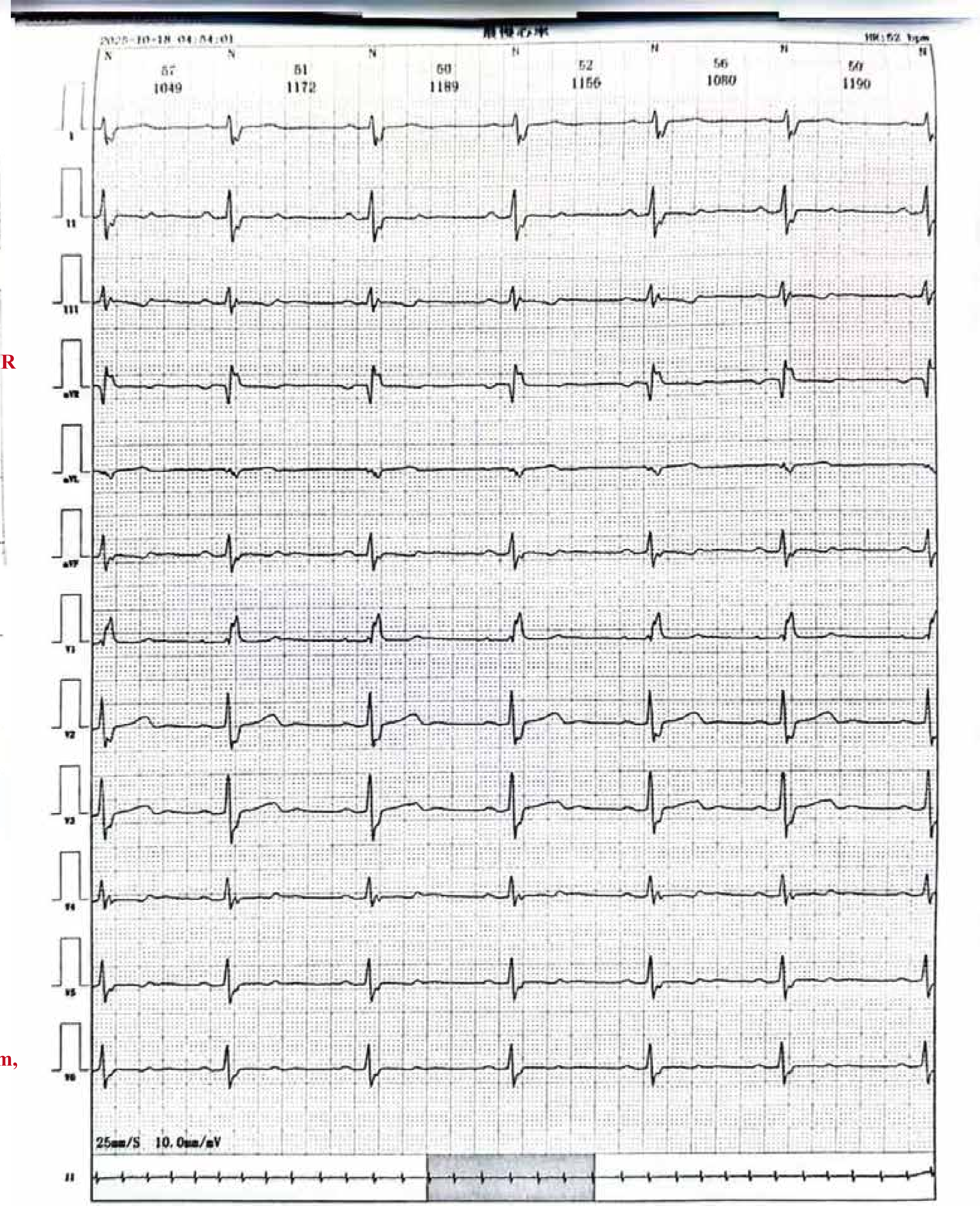

Supplement: Supplementary file 4 [file mmc4.pdf]
