# Supplementary material for: Focal pulsed-field ablation for recurrent para-Hisian premature ventricular contractions after failed radiofrequency ablation: A case report
Source: HeartRhythm Case Rep. 2026 Jun 22;12(9):1148–51. doi: 10.1016/j.hrcr.2026.06.017 (PMC13593549; doi:10.1016/j.hrcr.2026.06.017)

动态心电图报告

患者信息

姓名:	病区与床号:	申请号:
病人ID:	住院/体检/门诊号:	申请科室:
性别:	检查医生:	用药:
年龄:	记录时间:2026-05-12	临床诊断:

心率

总心搏数:116404

记录时长:23:20:18 **Recording Duration: 23 h 20 min**

平均心率:83 bpm **Mean HR**

最快心率:121 bpm 发生在 05-13 08:02:37 长RR间期 (>2000 ms):0 次 **Max HR**

最慢心率:54 bpm 发生在 05-13 00:49:52 **Min HR**

室早

总数:0 **PVCs**	平均(小时):
单发:0	成对:0
二联律:0 阵	三联律:0 阵
R on T:0	室速总数:0
插入性室早:0	
最长的室速:0 次	
最快心率的室速:--	
最慢心率的室速:—	

房早

总数: 1 (小于 1 %)	平均(小时):0
单发:1	成对:0
二联律:0 阵	三联律:0 阵
房早伴差传:0	房早未下传:0
房速:0	
最长的房速:0 次	
最快心率的房速:—	
最慢心率的房速:—	

逸博

室性逸搏:0 房性逸搏:0

交界性

交界性早搏:0 交界性逸搏:0

HRV

SDNN:119 (ms) SDNN Idx:37 (ms) rMSSD:20 (ms)

SDANN:116 (ms) pNN50:2% 三角指数:24

VLF:783.64(ms2) LF:315.72(ms2) HF:151.88(ms2)

ST段分析

最大压低 —

最大抬高 —

房颤/房扑

房颤心搏:0

房扑心搏:0

起搏 (未分析)

起搏心搏:—	房性起搏:—
室性起搏:—	双腔起搏:—

结论 **Conclusions**

平均心率是 83bpm。最慢心率是 54bpm，发生于 05-13 00:49:52。最快心率是 121bpm，发生于 05-13 08:02:37 。共分析心搏总数 116404次。
房性早搏共有 1次，单发房早有1次。
未检测到室性早搏发生。
未见大于 2000ms的长RR事件。

1、窦性心律
2、偶发房性早搏
3、完全性右束支传导阻滞

Quantitative Analysis:
Mean heart rate: 83 bpm
Minimum heart rate: 54 bpm
Maximum heart rate: 121 bpm
Total beats: 116,404
1 premature atrial contraction (PAC)
No premature ventricular contractions (PVCs) detected.
No RR intervals > 2 seconds were observed.

1.Sinus rhythm.
2.Isolated premature atrial contraction (PAC).
3.Complete right bundle branch block

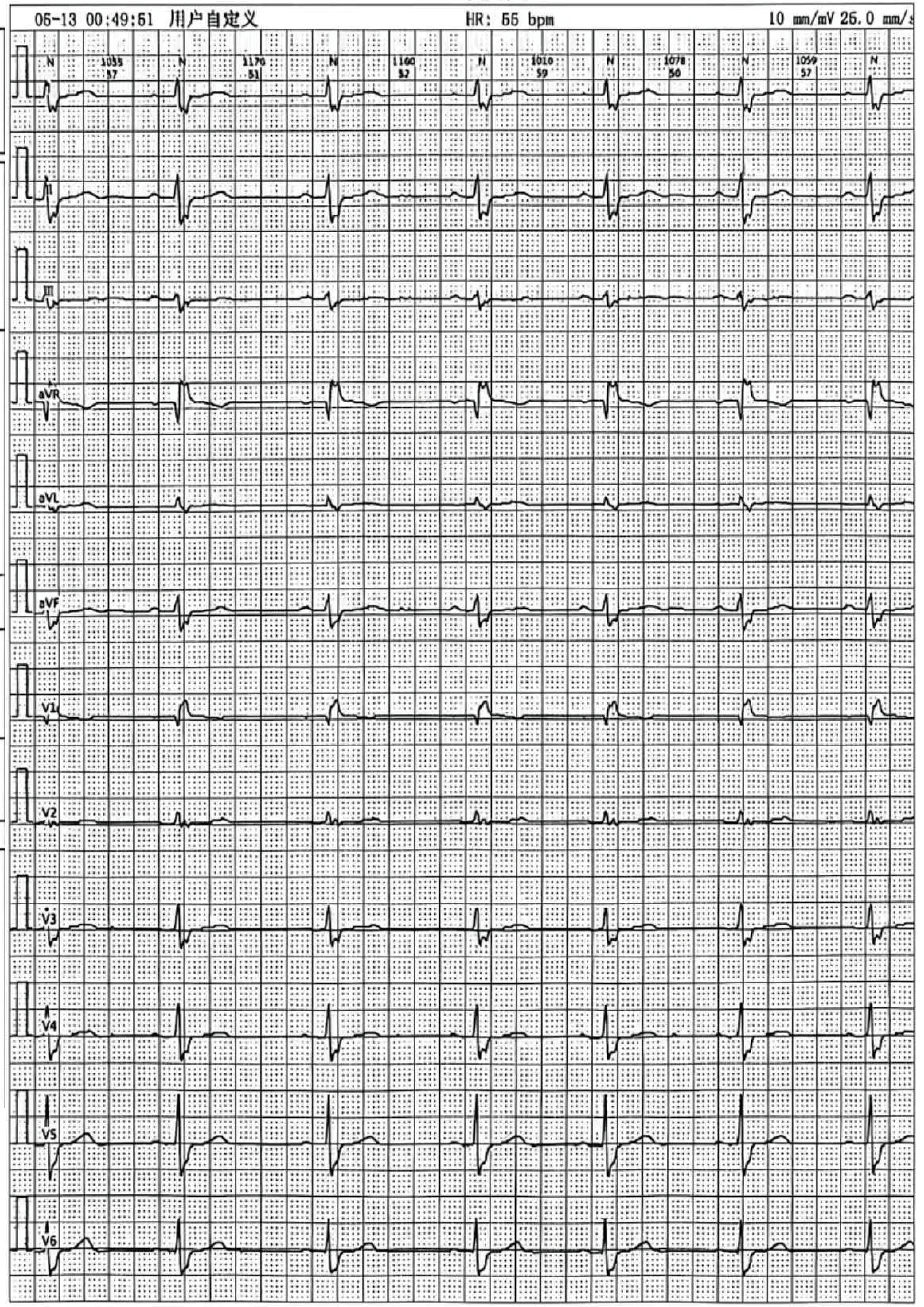

Supplement: Supplementary file 5 [file mmc5.pdf]
