# Supplementary material for: Focal pulsed-field ablation for recurrent para-Hisian premature ventricular contractions after failed radiofrequency ablation: A case report
Source: HeartRhythm Case Rep. 2026 Jun 22;12(9):1148–51. doi: 10.1016/j.hrcr.2026.06.017 (PMC13593549; doi:10.1016/j.hrcr.2026.06.017)
Supplement: Supplementary file 6 [file mmc6.docx]

Supplementary 1. Holter Monitoring Data

A. Preoperative 24-h Holter: PVC burden 38.8%.

B. 1-month post-RFA Holter: PVC burden 33.5%.

Supplementary 2. Serial 12-lead ECG Evolution

A. Admission ECG: RVOT posteroseptal PVC morphology.

B. Post-ablation monitoring: Sinus rhythm without PVC recurrence.

C. Pre-discharge ECG: Stable sinus rhythm (PR 174 ms).

D. The 1-month follow-up: Absent PVCs with PR 172 ms.

F. The 6-month follow-up: Absent PVCs with PR 189 ms.

Supplementary 3. Intracardiac Electrograms

A. Effective site: Intermittent His potential preceding local ventricular signal.

B. Recovery after transient AV dissociation: Transition to sinus rhythm with PR interval measurement (198 ms).

C. Recovery after third-degree AV block: Restoration of conduction with PR interval measurement (187 ms).

D. Newly emerged RBBB: First observed during catheter manipulation (before PFA).

Supplementary 4. 24-Hour Holter Monitoring Results at 1-Month Follow-Up

This summary report demonstrates a complete absence of premature ventricular contractions with a 0% overall burden one month after the focal pulsed-field ablation procedure.

Supplementary 5. 24-Hour Holter Monitoring Results at 8-Month Follow-Up

This summary report demonstrates a complete absence of premature ventricular contractions with a 0% overall burden at eight-month follow-up after the focal pulsed-field ablation procedure.
